# A phase II trial of paclitaxel and epirubicin in advanced breast cancer

**DOI:** 10.1054/bjoc.2000.1306

**Published:** 2000-07-24

**Authors:** D Rischin, J Smith, M Millward, C Lewis, M Boyer, G Richardson, G Toner, H Gurney, J McKendrick

**Affiliations:** Division of Haematology and Medical Oncology, Statistical Centre, Peter MacCallum Cancer Institute, Melbourne; Department of Medical Oncology, Prince of Wales Hospital, Sydney; Department of Medical Oncology, Royal Prince Alfred Hospital, Sydney; Department of Medical Oncology, Monash Medical Centre, Melbourne; Department of Medical Oncology, Westmead Hospital, Sydney; Department of Medical Oncology, Box Hill Hospital, Melbourne, Australia

**Keywords:** taxanes, anthracyclines, breast cancer, phase II

## Abstract

Initial trials of paclitaxel and doxorubicin in advanced breast cancer yielded high response rates but significant cardiac toxicity was observed. In this phase II trial we investigated the efficacy and safety of paclitaxel combined with epirubicin. Patients with advanced breast cancer, performance status 0–2, measurable disease, and a normal left ventricular ejection fraction, who may have received adjuvant chemotherapy were treated with epirubicin 75 mg m^–2^followed by a 3-h infusion of paclitaxel 175 mg m^–2^repeated every 3 weeks. Forty-three eligible patients were treated at six centres. 67% patients received the maximum of six cycles. The response rate was 54% (95% CI 38–69%), 12% CR and 42% PR. Estimated median progression-free survival was 6.9 months (95% CI 5.4–10.0) and estimated median overall survival was 17.9 months (95% CI 14.2–25.7). Four patients had a decrease in the left ventricular ejection fraction (LVEF) of ≥20% of baseline value, and in two patients the LVEF decreased to below the lower limit of normal, but no patient developed clinical evidence of cardiac failure. Grade 4 neutropenia occurred in 56% cycles, but only 4% of cycles were complicated by febrile neutropenia. Grade 3 or 4 non-haematologic toxicity was uncommon. In conclusion, paclitaxel 175 mg m^–2^and epirubicin 75 mg m^–2^is a well tolerated, promising regimen for the treatment of advanced breast cancer. © 2000 Cancer Research Campaign


*British Journal of Cancer* (2000) **83**(4), 438–442
© 2000 Cancer Research Campaign
doi: 10.1054/ bjoc.2000.1306, available online at http://www.idealibrary.com on IDEAL®

### A phase II trial of paclitaxel and epirubicin in advanced breast cancer

**D Rischin[1], J Smith[2], M Millward[1], C Lewis[3], M Boyer[4], G Richardson[5], G Toner[1], H Gurney[6] and J McKendrick[7]**

[1]Division of Haematology and Medical Oncology; [2]Statistical Centre, Peter MacCallum Cancer Institute, Melbourne; [3]Department of Medical Oncology, Prince of Wales Hospital, Sydney; [4]Department of Medical Oncology, Royal Prince Alfred Hospital, Sydney; [5]Department of Medical Oncology, Monash Medical Centre, Melbourne; [6]Department of Medical Oncology, Westmead Hospital, Sydney; [7]Department of Medical Oncology, Box Hill Hospital, Melbourne, Australia

**Summary** Initial trials of paclitaxel and doxorubicin in advanced breast cancer yielded high response rates but significant cardiac toxicity was observed. In this phase II trial we investigated the efficacy and safety of paclitaxel combined with epirubicin. Patients with advanced breast cancer, performance status 0–2, measurable disease, and a normal left ventricular ejection fraction, who may have received adjuvant chemotherapy were treated with epirubicin 75 mg $m^{-2}$ followed by a 3-h infusion of paclitaxel 175 mg $m^{-2}$ repeated every 3 weeks. Forty-three eligible patients were treated at six centres. 67% patients received the maximum of six cycles. The response rate was 54% (95% CI 38–69%), 12% CR and 42% PR. Estimated median progression-free survival was 6.9 months (95% CI 5.4–10.0) and estimated median overall survival was 17.9 months (95% CI 14.2–25.7). Four patients had a decrease in the left ventricular ejection fraction (LVEF) of ≥20% of baseline value, and in two patients the LVEF decreased to below the lower limit of normal, but no patient developed clinical evidence of cardiac failure. Grade 4 neutropenia occurred in 56% cycles, but only 4% of cycles were complicated by febrile neutropenia. Grade 3 or 4 non-haematologic toxicity was uncommon. In conclusion, paclitaxel 175 mg $m^{-2}$ and epirubicin 75 mg $m^{-2}$ is a well tolerated, promising regimen for the treatment of advanced breast cancer. © 2000 Cancer Research Campaign

**Keywords:** taxanes; anthracyclines; breast cancer; phase II

There has been considerable interest in determining the optimal way to combine the taxanes with anthracyclines, as these are the most active anti-cancer agents available for the treatment of advanced breast cancer (Hortobagyi and Holmes, 1997). Initial trials of 3-hour infusions of paclitaxel combined with doxorubicin reported high response rates, but significant cardiac toxicity (Gianni et al, 1995; Gehl et al, 1996). Epirubicin has similar efficacy to doxorubicin in breast cancer, with less cardiac toxicity (Bonadonna et al, 1993). Hence, combining paclitaxel with epirubicin may be one way of decreasing the risk of cardiac toxicity while maintaining efficacy. We have previously reported the results of a single-institution phase I trial of this combination in minimally pretreated patients, where the doses were escalated from epirubicin 60 mg $m^{-2}$ and paclitaxel 155 mg $m^{-2}$ to 90 mg $m^{-2}$ and 200 mg $m^{-2}$ respectively (Rischin et al, 1999). The dose-limiting toxicities were febrile neutropenia, oesophagitis and diarrhoea, and the recommended phase II doses were epirubicin 75 mg $m^{-2}$ and paclitaxel 175 mg $m^{-2}$. In the current trial we have investigated the combination of paclitaxel and epirubicin in advanced breast cancer, using the recommended doses from our phase I trial.

### PATIENTS AND METHODS

### Eligibility

Patients were required to have histologically proven advanced breast cancer. Patients may have received adjuvant chemotherapy but no prior chemotherapy for advanced disease. Prior anthracycline was permitted if at least 12 months had elapsed between completion of adjuvant treatment and relapse, and total doxorubicin dose was ≤240 mg $m^{-2}$. No prior taxane chemotherapy was permitted. Prior radiotherapy to less than 25% of the marrow-bearing areas was permitted, but radiotherapy must have been completed at least 4 weeks prior to study entry. Patients had to have a bidimensionally measurable lesion with at least one diameter >1 cm. Other eligibility criteria were: age 18–75 years, performance status (ECOG) 0–2, absolute neutrophil count ≥ 2.0 × $10^9$ $l^{-1}$, platelet count ≥ 100 × $10^9$ $l^{-1}$, bilirubin ≤ upper limit of normal, transaminases ≤2 times upper limit of normal, serum creatinine ≤ 1.5 times upper limit of normal and left ventricular ejection fraction (LVEF) measured by radionuclide ventriculography ≥ lower limit of normal. Written informed consent was obtained from all patients and the protocol was approved by the Institutional Ethics Committees.

Patients were excluded from the trial for any of the following: history of atrial or ventricular arrhythmias or cardiac failure, myocardial infarction within the preceding 6 months, history of second- or third-degree heart block, brain or bone metastases as the only known sites of disease, known bone-marrow metastases, pre-existing peripheral neuropathy > grade 2 by the Common Toxicity Criteria of the National Cancer Institute, history of other malignancy except for non-melanoma skin cancer or carcinoma in situ of the cervix, and pregnancy or lactation.

The phase I and II trials were included in the one protocol, with additional eligibility criteria for the phase II component including a diagnosis of advanced breast cancer, bidimensionally measurable disease and no prior chemotherapy for advanced disease. Patients treated at the recommended dose level on the phase I trial

*Received 17 November 1999*
*Revised 28 February 2000*
*Accepted 13 April 2000*

*Correspondence to:* D Rischin who met the eligibility requirements for the subsequent phase II trial were to be included in the phase II analysis.

### Pretreatment and follow-up evaluations

Before enrolment all patients underwent a full history, physical examination, complete blood count (CBC) with differential, electrolytes, liver function tests, creatinine, ECG, LVEF, and imaging of known sites of disease. While on study, patients were clinically assessed for toxicity weekly during the first cycle then every cycle subsequently. CBC including differential was performed weekly throughout treatment (twice weekly during first cycle) and electrolytes, creatinine and liver function tests were performed before each cycle. CT scanning and imaging of known sites of disease were performed every 2 cycles, as were gated cardiac scans.

Toxicity from treatment was graded according to the National Cancer Institute Common Toxicity Criteria (NCI-CTC). Anti-tumour activity was assessed according to the WHO response criteria.

### Doses and schedule

All patients received epirubicin 75 mg $m^{-2}$ followed by paclitaxel 175 mg $m^{-2}$ as a 3-h infusion, which were the recommended doses from our phase I trial. Cycles were repeated every 3 weeks. Dose escalation was not permitted in individual patients and the minimum interval between treatment cycles was 21 days. As the potential incidence of cardiac toxicity could not be accurately determined from phase I trials, it was decided to limit the number of cycles to a maximum of six. Patients with progressive disease were taken off study.

### Drug administration

Paclitaxel (Anzatax™) was supplied by Faulding Hospital Pharmaceuticals (Melbourne, Australia) as a sterile solution in polyethoxylated castor oil (Cremophor EL) and ethanol (50:50 v/v). Epirubicin (Pharmorubicin) was obtained from Pharmacia-Upjohn (Sydney, Australia). Epirubicin was administered first over 15–20 min followed immediately by a 3-h infusion of paclitaxel. Paclitaxel was diluted in 500 ml dextrose 5% and stored in a sterile glass container and administered through polyethylene-lined tubing with a cellulose acetate 0.22-μm in-line filter. Prior to every cycle patients were premedicated with dexamethasone 20 mg orally, 12 and 6 h before the paclitaxel infusion, and cimetidine 300 mg (alternatively ranitidine 50 mg) and promethazine 25 mg both intravenously, 30 min prior to the paclitaxel infusion. Prophylactic recombinant granulocyte colony stimulating factor (G-CSF) support was not permitted.

### Dose modifications for toxicity

Dose reductions were defined for haematologic and non-haematologic toxicities. Epirubicin and paclitaxel doses were to be reduced by 25% for febrile neutropenia, nadir neutrophil count $<0.5 \times 10^9$ $l^{-1}$ for ≥7 days, or nadir platelet count $<50\times10^9$ $l^{-1}$. No dose modification was made for uncomplicated neutrophil count $<0.5\times10^9$ $l^{-1}$ for <7 days. If the day 21 neutrophil count was $<1.5 \times 10^9$ $l^{-1}$, or the platelet count $<100 \times 10^9$ $l^{-1}$, further treatment was delayed weekly until recovery. WHO grade 3 or 4 non-haematologic toxicity were generally managed by a 25% dose reduction of both drugs. However, patients could be managed by symptomatic treatment alone or removal from the study at the investigator's discretion. Clinically significant hypersensitivity reactions (defined as hypotension that required therapy, angioedema, respiratory distress requiring bronchodilator therapy, or generalized urticaria) required cessation of the paclitaxel infusion and appropriate supportive measures.

### Statistics

The primary outcome measure was response rate, with secondary outcomes including progression-free and overall survival and toxicity. 95% confidence intervals for the response rates were estimated using the exact probabilities of the binomial distribution (StatXact, 1999). The close-out date for survival analyses was October 1, 1998. The median potential follow-up time from commencing treatment to the close-out date was 22 months, range 11.5–43 months. Overall survival and progression-free survival from the commencement of protocol treatment were estimated using the Kaplan–Meier product-limit method. For overall survival, all deaths were counted regardless of cause and survival times for living patients were censored at the close-out date. For progression-free survival, progression at any site or death from any cause was counted as an event, with censoring at the close-out date for patients surviving without progression. The Brookmeyer–Crowley method was used to estimate 95% confidence intervals for median survival times (S-plus, 1997).

### RESULTS

### Patient characteristics

Forty-five patients from six Australian centres were enrolled in this trial between March 1995 and October 1997. Two patients were subsequently found to be ineligible due to prior chemotherapy for metastatic disease. The details of the remaining 43 patients enrolled on this study are given in Table 1. Five of these patients were treated on the phase I study (Rischin et al, 1999), and also included in this analysis as originally intended, as they were treated at the recommended dose level and fulfilled all the eligibility requirements for the phase II trial.

### Treatment

Two hundred and sixteen cycles of treatment (median 6, range 2–6) were given. Sixty-seven percent of patients received all six cycles. Reasons for ceasing treatment prior to six cycles were: progressive disease 21%, toxicity 7%, death 2% and patient request 2%. The toxicities that resulted in treatment cessation were: fatigue, reduced left ventricular ejection fraction and treating physician decision to withdraw a patient with a urinary tract infection and neutropenia. The death was the result of a non-neutropenic chest infection 3 days after receiving the fifth cycle. Eighteen (42%) patients required 22 dose reductions, predominantly for low blood counts and febrile neutropenia.

### Toxicity

Worst toxicities experienced during treatment are given in Table 2. Neutropenia was the predominant toxicity, with 95% of patients

*© 2000 Cancer Research Campaign*

**Table 1** Patient characteristics ($n = 43$)

| | |
|---|---|
| Median age (range) | 52 years (35–70) |
| Performance status (ECOG) | |
| 0 | 18 |
| 1 | 21 |
| 2 | 4 |
| Adjuvant chemotherapy | |
| None | 24 |
| CMF(P) | 16 |
| Anthracycline + CMF | 3 |
| Prior hormonal therapy | 28 |
| Prior radiotherapy | 25 |
| Disease sites | |
| Visceral +/– bone +/– soft tissue | 31 |
| Bone +/– soft tissue | 6 |
| Soft tissue only | 6 |

experiencing grade 4 neutropenia (121/216 cycles). However, febrile neutropenia only occurred in six (14%) patients, and eight (4%) cycles. Grade 4 neutropenia lasting ≥7 days occurred in four patients, one cycle each. Grade 3 or 4 non-haematologic toxicity was uncommon. Four patients required dose delays due to slow recovery from myelosuppression, with one patient requiring a dose delay on two occasions.

The median cumulative dose of epirubicin was 371 mg $m^{-2}$ (range 131–459). Four patients had a decrease in the left ventricular ejection fraction (LVEF) of ≥20% of baseline value, and in two patients the LVEF decreased to below the lower limit of normal, but no patient developed clinical evidence of cardiac failure. None of these four patients had received prior anthracyclines. In the three patients who had previously received doxorubicin the LVEF remained within the normal range and the decrease in LVEF was <20%. In 20 patients who received ≥400 mg $m^{-2}$ epirubicin the mean change in LVEF, expressed as a percentage of the baseline value, was a decrease of 11%.

### Responses

Five patients (12%) achieved a complete response and 18 (42%) a partial response to give an overall response rate of 54% (95% CI 38–69%). Thirteen patients (30%) had stable disease and seven (16%) had progressive disease. Of the five complete responders, two had visceral disease, two nodal involvement and one had chest-wall disease. The response rate in patients who had received adjuvant chemotherapy was not significantly different from that in patients who had received no prior chemotherapy, 53% and 54% respectively. One of the three patients who had received prior anthracycline achieved a partial response.

**Table 2** Toxicity

| | Worst grade[a] (percentage of patients) | | | | |
|---|---|---|---|---|---|
| | 0 | 1 | 2 | 3 | 4 |
| Haemoglobin | 12 | 42 | 40 | 7 | 0 |
| Neutrophils | 0 | 0 | 2 | 2 | 95 |
| Platelets | 47 | 42 | 5 | 5 | 2 |
| Stomatitis | 56 | 14 | 26 | 5 | 0 |
| Nausea | 33 | 42 | 19 | 7 | – |
| Vomiting | 56 | 19 | 23 | 2 | 0 |
| Diarrhoea | 77 | 14 | 5 | 2 | 2 |
| Sensory neuropathy | 42 | 44 | 14 | 0 | – |
| Motor neuropathy | 98 | 2 | 0 | 0 | 0 |
| Cardiac function | 93 | 2 | 5 | 0 | 0 |
| Alopecia | 2 | 2 | 95 | – | – |
| Infection | 70 | 0 | 12 | 19 | – |
| Myalgia/arthralgia | 28 | 33 | 26 | 14 | – |
| Asthenia | 37 | 30 | 21 | 12 | – |

[a]For non-haematological toxicities, worst grade considered to be related to study drugs

### Survival

By the close-out date 34 patients (79%) had progressed or relapsed and 22 (51%) had died of their disease. One additional patient (2%) died without progression due to a chest infection. Progression-free survival is demonstrated in Figure 1. The estimated median progression-free survival is 6.9 months (95% CI 5.4–10.0). Overall survival is demonstrated in Figure 2. The estimated median survival is 17.9 months (95% CI 14.2–25.7). No second-line treatment was specified in the protocol. Of the 34 patients who progressed, seven were given no further systemic

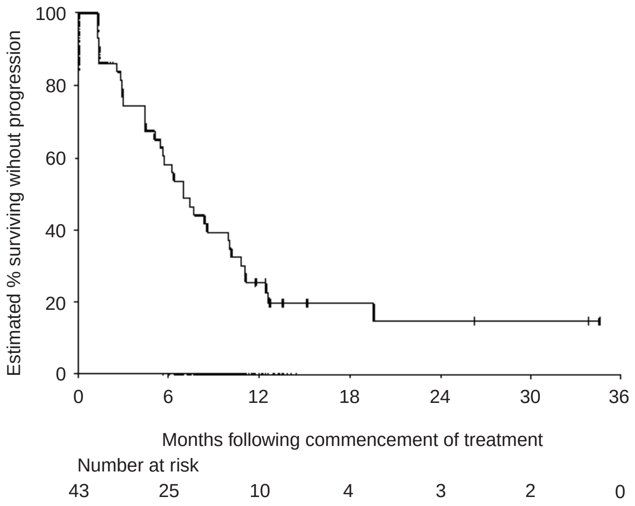

**Figure 1** Progression-free survival of all patients. Vertical marks indicate censoring for patients surviving progression-free at the close-out date

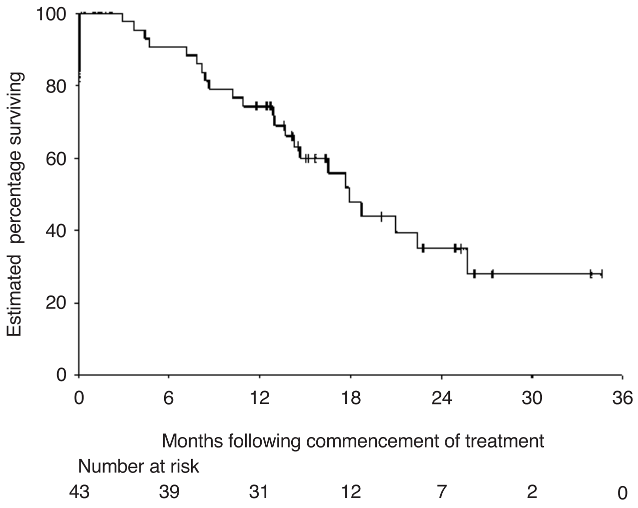

**Figure 2** Overall survival of all patients. Vertical marks indicate censoring for patients surviving at the close-out date

*© 2000 Cancer Research Campaign*

treatment prior to the close-out date, four received radiotherapy alone, four received hormonal therapy and 19 received second-line chemotherapy. This consisted of cyclophosphamide, methotrexate and 5-Fluorouracil in 13 cases and various regimens in the other six. Data was not collected about responses to these treatments.

### DISCUSSION

This multi-centre phase II trial has demonstrated that the combination of paclitaxel and epirubicin is a promising regimen, achieving an objective response rate of 54% and median survival of 17.9 months, with an acceptable toxicity profile. Previous trials with paclitaxel and doxorubicin have reported response rates of 47–94% (Gianni et al, 1995; Hortobagyi and Holmes, 1997; Sledge et al, 1998). Conte et al (1997) have reported a response rate of 84% with paclitaxel and epirubicin, but other trials have reported response rates of 43–68% (Carmichael et al, 1997; Catimel et al, 1997; Luck et al, 1997). Differences in patient populations treated may partly account for differences in activity observed. Furthermore, there were differences in trial design between the trial reported by Conte et al (1997) and our trial.. In particular, Conte used higher doses of paclitaxel and epirubicin, gave prophylactic ciprofloxacin and fluconazole to all patients and permitted the use of G-CSF at all dose levels to shorten the duration of grade 4 neutropenia or in the event of febrile neutropenia. The use of higher doses of epirubicin and paclitaxel with G-CSF resulted in a higher dose intensity than was achieved in our trial, and this may have contributed to the difference in response rates between the two trials. However, we have previously reported that the addition of G-CSF did not permit any further dose escalation in our phase I trial of paclitaxel and epirubicin (Rischin et al, 1999), hence our decision not to use G-CSF in the phase II trial. The role of G-CSF in patients with advanced breast cancer receiving conventional dose chemotherapy remains unproven.

In view of the promising phase II results achieved with anthracycline and taxane combinations in advanced breast cancer, several phase III trials are addressing the role of such combinations in breast cancer in both the adjuvant and advanced disease settings (Conte and Gennari, 1997). Sledge et al (1997) reported a higher response rate and longer time to treatment failure with paclitaxel (24-h infusion) and doxorubicin compared to single-agent paclitaxel or single-agent doxorubicin, but there was no significant difference in overall survival (Sledge et al, 1998). It remains unclear whether such combinations are superior to the sequential use of taxanes and anthracyclines, but ongoing trials should assist in resolving this question. Paclitaxel and anthracycline combinations have not been compared directly to docetaxel and anthracycline combinations in randomized trials. In a preliminary report the combination of docetaxel and doxorubicin resulted in a superior response rate compared to the combination of doxorubicin and cyclophosphamide (Nabholtz et al, 1999).

No cases of congestive cardiac failure were observed in our phase I trial, the current phase II trial, or in the trial of Conte et al (1997). However, patients received a maximum of six cycles on this trial, and hence the median cumulative dose of epirubicin was relatively low at 371 mg $m^{-2}$. Cardiotoxicity was a major complication in initial trials combining 3-h infusions of paclitaxel with doxorubicin, with a 20% incidence of congestive cardiac failure reported (Gianni et al, 1995; Gehl et al, 1996). Pharmacokinetic studies have demonstrated increased plasma concentrations of doxorubicin and/or doxorubicinol when doxorubicin is given with paclitaxel (Holmes et al, 1996; Gianni et al, 1997*a*; Berg et al, 1994), and this may contribute to the increased risk of cardiac toxicity. Paclitaxel is formulated in 50% Cremophor EL (cremophor), which can result in similar alterations to doxorubicin pharmacokinetics when administered without paclitaxel (Millward et al, 1998). Epirubicin has similar efficacy with less cardiotoxicity than doxorubicin, and the pharmacokinetic interaction with paclitaxel may be less clinically significant than with doxorubicin (Conte and Gennari, 1997; Gianni et al, 1997*b*; Rischin et al, 1999). The lower risk of cardiac toxicity may be a potential advantage for combination chemotherapy with paclitaxel, and has provided the impetus for current phase III trials which are investigating the role of paclitaxel and epirubicin in the treatment of both early and advanced breast cancer.

### ACKNOWLEDGEMENTS

Trial was supported in part by Faulding Pharmaceuticals and Pharmacia-Upjohn.

### REFERENCES

Berg SL, Cowan KH, Bolis FM, Fisherman JS, Denicoff AM, Hillig M, Poplack DG and O'Shaughnessy JA (1994) Pharmacokinetics of taxol and doxorubicin administered alone and in combination by continuous 72 hour infusion. *J Natl Cancer Inst* **86**(2): 143–145

Bonadonna G, Gianni L, Santoro A, Bonfante V, Bidoli P, Casali P, Demicheli R and Valagussa P (1993) Drugs ten years later: Epirubicin. *Ann Oncol* **4**: 359–369

Carmichael J, Jones A and Hutchinson T (1997) A phase II trial of epirubicin plus paclitaxel in metastatic breast cancer. *Semin Oncol* **24**(5,17): S17-44–S17–47

Catimel G, Spielmann M, Dieras V, Tubiana-hulin M, Bonneterre J, Pouillart P, Kayitalire L, Guastalla JP, Graffand Ni, Garet F, Dumortier A and Pellae-Cosset B (1997) Phase I studies of combined paclitaxel/epirubicin and paclitaxel/epirubicin/cyclophosphamide in patients with metastatic breast cancer: the French experience. *Semin Oncol* **24**(1,3): S8–12

Conte PF, Baldini E, Gennari A, Michelotti A, Salvadori B, Tibaldi C, Danesi R, Innocenti F, Gentile A, Dell'Anna R, Biadi O, Mariani M and Del Tacca M (1997) Dose-finding study and pharmacokinetics of epirubicin and paclitaxel over 3 hours: A regimen with high activity and low cardiotoxicity in advanced breast cancer. *J Clin Oncol* **15**(7): 2510–2517

Conte PF and Gennari A (1997) Anthracyclines-paclitaxel combinations in the treatment of breast cancer. *Ann Oncology* **8**: 939–943

Gehl J, Boesgard M, Paaske T, Jensen V and Dombernowsky P (1996) Combined doxorubicin and paclitaxel in advanced breast cancer: effective and cardiotoxic. *Ann Oncol* **7**: 687–693

Gianni L, Munzone E, Capri G, Fulfaro F, Tarenzi E, Villani F, Spreafico C, Laffranchi A, Caraceni A, Martini C, Stefanelli M, Valagussa P and Bonadonna G (1995) Paclitaxel by 3 hour infusion in combination with bolus doxorubicin in women with untreated metastatic breast cancer: high antitumour efficacy and cardiac effects in a dose-finding and sequence-finding study. *J Clin Oncol* **13**(11): 2688–2699

Gianni L, Vigano L, Locatelli A, Capri G, Giani A, Tarenzi E and Bonadonna G (1997*a*) Human pharmacokinetic characterisation and in-vitro study of the interaction between doxorubicin and paclitaxel in patients with breast cancer. *J Clin Oncol* **15**(5): 1906–1915

Gianni L, Bigano L, Locatelli A, Giani A, Capri G, Bertuzzi A, Grasselli G, Tarenzi E and Bonadonna G (1997*b*) Different interference of paclitaxel (PTX) on human pharmacokinetics of doxorubicin (DOX) and epirubicin (EPI). *Proc Am Soc Clin Oncol* **16**: 786

Holmes FA, Madden T, Newman RA, Valero V, Theriault RL, Fraschini G, Walters RS, Booser DJ, Buzdar AU, Willey J and Hortobagyi GN (1996) Sequence-dependent alteration of doxorubicin pharmacokinetics by paclitaxel in a phase I study of paclitaxel and doxorubicin in patients with metastatic breast cancer. *J Clin Oncol* **14**: 2713–2721

Hortobagyi G and Holmes FA (1997) Optimal dosing of paclitaxel and doxorubicin in metastatic breast cancer. *Semin Oncol* **24**(1,3): S3–4–S3–7

© 2000 Cancer Research Campaign

Luck HJ, Thomssen C, du Bois A, Untch M, Lisboa BW, Kohler B and Diegarten K (1997) Metastatic breast cancer: experience with the combination epirubicin plus paclitaxel. *Oncology (Huntingt)* **12**(1): 36–39

Millward MJ, Webster LK, Rischin D, Stokes KH, Toner GC, Bishop JF, Olver IN, Linahan BM, Linsenmeyer ME and Woodcock DM (1998) Phase I trial of Cremophor EL with bolus doxorubicin. *Clin Cancer Res* **4**(10): 2321–2329

Nabholtz JM, Falkson G, Campos D, Szanto J, Martin M, Chan S, Pienkowski T, Bezwoda WR, Zaluski J, Pinter T, Krzakowski M, Vorobiof D, Leonard R, Kennedy I, Azli N, Murawsky M, Riva A and Pouillart P (1999) *Proc Am Soc Clin Oncol* **18**: 85

Rischin D, Webster L, Millward M, Toner G, Nawaratne S, Ganju V, Francis P and Bishop J (1999) A phase I and pharmacokinetic study of paclitaxel and epirubicin in advanced cancer. *Inv New Drugs* **17**: 73–80

Sledge GW Jr, Neuberg D, Ingle J, Martino S and Wood W (1997) Phase III trial of doxorubicin (A) vs paclitaxel (T) vs doxorubicin + paclitaxel (A + T) as first-line therapy for metastatic breast cancer (MBC): an intergroup trial. *Proc Am Soc Clin Oncol* **16**: 2

S-plus 4.0.3. (1997) MathSoft Inc: Seattle, WA

StatXact 4.0.1. (1999) Cytel Software Corporation: Cambridge, MA

*© 2000 Cancer Research Campaign*

## References

[PDF_00416] Berg S. L., Cowan K. H., Balis F. M., Fisherman J. S., Denicoff A. M., Hillig M., Poplack D. G., O'Shaughnessy J. A. (1994). Pharmacokinetics of taxol and doxorubicin administered alone and in combination by continuous 72-hour infusion.. J Natl Cancer Inst.

[PDF_00420] Bonadonna G., Gianni L., Santoro A., Bonfante V., Bidoli P., Casali P., Demicheli R., Valagussa P. (1993). Drugs ten years later: epirubicin.. Ann Oncol.

[PDF_00429] Conte P. F., Baldini E., Gennari A., Michelotti A., Salvadori B., Tibaldi C., Danesi R., Innocenti F., Gentile A., Dell'Anna R. (1997). Dose-finding study and pharmacokinetics of epirubicin and paclitaxel over 3 hours: a regimen with high activity and low cardiotoxicity in advanced breast cancer.. J Clin Oncol.

[PDF_00434] Conte P. F., Gennari A. (1997). Anthracyclines-paclitaxel combinations in the treatment of breast cancer.. Ann Oncol.

[PDF_00436] Gehl J., Boesgaard M., Paaske T., Vittrup Jensen B., Dombernowsky P. (1996). Combined doxorubicin and paclitaxel in advanced breast cancer: effective and cardiotoxic.. Ann Oncol.

[PDF_00439] Gianni L., Munzone E., Capri G., Fulfaro F., Tarenzi E., Villani F., Spreafico C., Laffranchi A., Caraceni A., Martini C. (1995). Paclitaxel by 3-hour infusion in combination with bolus doxorubicin in women with untreated metastatic breast cancer: high antitumor efficacy and cardiac effects in a dose-finding and sequence-finding study.. J Clin Oncol.

[PDF_00445] Gianni L., Viganò L., Locatelli A., Capri G., Giani A., Tarenzi E., Bonadonna G. (1997). Human pharmacokinetic characterization and in vitro study of the interaction between doxorubicin and paclitaxel in patients with breast cancer.. J Clin Oncol.

[PDF_00453] Holmes F. A., Madden T., Newman R. A., Valero V., Theriault R. L., Fraschini G., Walters R. S., Booser D. J., Buzdar A. U., Willey J. (1996). Sequence-dependent alteration of doxorubicin pharmacokinetics by paclitaxel in a phase I study of paclitaxel and doxorubicin in patients with metastatic breast cancer.. J Clin Oncol.

[PDF_00462] Lück H. J., Thomssen C., Du Bois A., Untch M., Lisboa B., Köhler G., Diergarten K. (1998). Metastatic breast cancer: experience with the combination paclitaxel plus epirubicin.. Oncology (Williston Park).

[PDF_00465] Millward M. J., Webster L. K., Rischin D., Stokes K. H., Toner G. C., Bishop J. F., Olver I. N., Linahan B. M., Linsenmeyer M. E., Woodcock D. M. (1998). Phase I trial of cremophor EL with bolus doxorubicin.. Clin Cancer Res.

[PDF_00472] Rischin D., Webster L. K., Millward M. J., Toner G. C., Nawaratne S., Ganju V., Francis P., Bishop J. F. (1999). A phase I and pharmacokinetic study of paclitaxel and epirubicin in advanced cancer.. Invest New Drugs.

